# Amaurosis Following Dental Disease

**Published:** 1879-01

**Authors:** 


### AMAUROSIS FOLLOWING DENTAL DISEASE.

Sirletti (*Le Mouvement Med.* 1878, p. 193; from *Arch. Clin. Ital dei Med. Condetti*) reports the case of an old man attacked suddenly with hemoralopia, without any previous enfeeblement of vision. Knowing that dental troubles sometimes cause endo-ocular hyperæmia and amaurosis through the ophthalmic ganglion, S. inquired which of the patient's teeth were decayed. He found that for some time he had suffered severe pain in the upper canines, of which the crown was gone. On examination, a fistulous cavity, with intra and extra-alveolar periostitis, was found in the root of each canine. These teeth having been extracted, the patient began to be able to distinguish light from darkness almost immediately, and by the end of six days his sight was completely restored. —*Philadelphia Medical Times.*

